# Autoimmunity in Segmental Vitiligo

**DOI:** 10.3389/fimmu.2020.568447

**Published:** 2020-10-27

**Authors:** Reinhart Speeckaert, Jo Lambert, Vedrana Bulat, Arno Belpaire, Marijn Speeckaert, Nanja van Geel

**Affiliations:** ^1^ Department of Dermatology, Gent University Hospital, Gent, Belgium; ^2^ Department of Dermatology, University Hospital Centre Zagreb, Zagreb, Croatia; ^3^ Department of Nephrology, Gent University Hospital, Gent, Belgium

**Keywords:** segmental vitiligo, immunology, mosaicism, linear morphea, lichen striatus

## Abstract

The autoimmune basis of segmental vitiligo (SV) has only recently been recognized. Systemic autoimmune diseases are less frequently associated compared to non-segmental vitiligo (NSV), but localized skin disorders – in particular linear morphea – have been repeatedly observed in patients with SV. The inflammatory response is documented on a clinical level with cases displaying erythematous borders or a hypochromic stage, on a histopathological level with predominantly CD8 lymphocytes migrating toward the basal layer and by flow cytometry demonstrating the antimelanocyte specificity of these cytotoxic T cells. The increased risk for halo naevi and NSV in these patients further underline the immune-mediated mechanisms of SV. Nonetheless, the localized and unique distribution pattern points to somatic mosaicism. This places SV in a category of similar diseases such as lichen striatus, blaschkitis, linear lupus erythematosus, and linear scleroderma where an immune reaction against genetically mutated skin cells is believed to be the underlying cause. All these disorders are characterized by a young age of onset, a temporary disease activity with spontaneous resolution, limited response to treatment, and often long-term sequelae. Although challenging, genetic research proving this genetic mosaicism could offer crucial insights into the pathogenesis of both segmental and non-segmental vitiligo.

## Introduction

The prevalence of vitiligo is reported to be around 0.5-1% of the population, although remarkable regional differences exist (1). Segmental vitiligo (SV) accounts for 5-16% of all vitiligo cases and has a relatively equal gender distribution. However, some studies report a slight predominance of females (2, 3). SV has several unique characteristics compared to non-segmental vitiligo (NSV). SV usually involves only one body area and displays a sharp demarcation around the midline of the body. It can develop at all ages although a predominance around 4-10 years exists (4). Progression mostly occurs in the first year with a spontaneous stop of new depigmentations after 1.5-2 years in the majority of patients. In some patients, SV stabilizes even after a few days (8/141; 16.7%) (5). However, this is not always the case as in one study, 36.8% of patients still reported progression after 2 years (6). The development of SV areas on opposite body sites has been termed bilateral SV. Mixed vitiligo is the concomitant presence of both SV and NSV in the same patient (7). In contrast to NSV, poliosis is often visible at an early stage and far more frequent compared to NSV. When examined in detail, almost all SV patients exhibit leukotrichia (8). Itch might be an underrecognized symptom being present in 20% of SV patients (9).

Melanocytes carrying a mosaic mutation which elicits a targeted immune response are suspected as the underlying cause of SV (10). Additionally, several other hypotheses have been put forward in an attempt to explain SV including neurogenic mechanisms, genetic mosaicism, and oxidative stress. In the last years, research suggests that different theories likely play a combined role.

During the progressive phase, topical anti-inflammatory treatments including corticoids or immunomodulators are advised (11). UVB or excimer treatment can be initiated but the repigmentation rates are lower compared to NSV (12). Nonetheless, UVB/excimer therapy can be considered in patients with recent disease onset or combined with pigment cell transplantation. Shah et al. reported a 75% repigmentation in 78% of patients with 35.6% showing complete repigmentation after excimer light combined with topical tacrolimus (13). Both marginal and perifollicular repigmentation patterns can be observed (14).

## Auto-Immunity in Segmental Vitiligo

NSV is characterized by multiple auto-immune disorders with thyroid disease and alopecia areata being the most prevalent. The rates of auto-immune disorders are higher in NSV compared to SV, especially concerning thyroid disease (15). Lim et al. found positive thyroid peroxidase antibodies in 24.7% of NSV patients while this was only 15.1% in SV (16). A systematic review documented a prevalence of thyroid disorders of 3/69 (4.3%) in SV which was not increased compared to healthy controls (17). This underlines the difference in pathogenesis between both disorders. While in NSV, a genetic background of increased risk to autoimmunity has been uncovered by genome-wide analyses [e.g. a major histocompatibility complex (MHC) enhancer polymorphism], this is less expected in its segmental counterpart (18). A relatively small whole-genome expression study (20 SV; 20 NSV; 20 healthy controls) found more differentially expressed genes involved in the adaptive immune response in SV while in NSV regulation of the innate immune response, B cell differentiation and activation was more prominent (19). This suggests that the melanocyte immune response in SV is more targeted and involves a narrower inflammatory activation profile (e.g. less evidence for autophagy) compared to NSV. The inflammatory response in SV is short-lived and localized, but evidence for an immune-based cytotoxic destruction of SV melanocytes is increasing. From a clinical perspective, 2 cases of inflammatory SV have been reported (20–22). In a relatively small study, immunohistochemistry revealed a lesional lymphocytic infiltrate in 8/12 of progressing SV patients comparable to NSV with a predominance of CD8^+^ T cells migrating toward the basal layer. A non-significant increase in IFN-γ was observed (23). In a larger study (SV n = 50), histopathological inflammatory features were noticed in 78% (38/50) of patients. In 42% (21/50), small clusters of lymphocytes were found around melanocytes. In 36% (18/50) of SV patients, a focal lichenoid inflammation was present and in 22% (11/50) post-inflammatory changes were observed (24). Our group analyzed the lymphocytic infiltrate in a progressive case of SV more in-depth using HLA-peptide tetramers (MART-1, tyrosinase, gp100). We discovered both in lesional and non-lesional skin CD8 lymphocytes with a high production of IFN-γ and TNF-α. An increased percentage of CD8 lymphocytes targeting the melanocyte differentiation peptide gp100 was observed both in lesional and non-lesional skin (25).

Nonetheless, this antimelanocyte-specific response can explain why halo naevi can develop and why progression to NSV can occur. Deregulation of chemotactic signals of CCL11, CCL17, CCL22, CCL24, CCL27, CXCL10, and CXCL12 was found in SV which was associated with a prominent inflammatory infiltrate. CD11c+ dendritic cells were abundantly present together with CD8 T cells (26). In another study, no difference in lesional versus non-lesional SV levels of sICAM-1 and GM-CSF was detected (n=16) (27). However, this study did not specify whether it concerned stable or progressive SV vitiligo lesions which might explain the lack of difference in inflammatory markers. SV skin contains increased epidermal oxidative stress (H_2_O_2_/ONOO(-)) (28). Similar to NSV, increased levels of superoxide dismutase (SOD), decreased antioxidative enzymes such as catalase, and increased lipid peroxidation levels were found (29). Whether this concerns a primary cause or a secondary phenomenon due to inflammation, remains a topic of debate. Up till now, reproducible results of effective SV treatment with antioxidants are lacking.

Poliosis is an important and early sign of SV. This indicates that the immune-privileged environment around the hairs is less protective in SV compared to NSV. As the reservoir of melanocyte precursors around the hair bulb is often depleted in SV, repigmentation is less impressive when phototherapy is initiated (12). Several immune checkpoints [e.g. programmed death ligand-1 (PD-L1), indoleamine 2,3-dioxygenase (IDO)] have been found to regulate the immune privilege of the hair follicle. Inhibition of these factors by immunotherapy for melanoma results often in the development of vitiligo-like lesions very similar to NSV (30, 31). It is therefore plausible to speculate that SV is less dependent on changes in factors regulating immune privilege compared to NSV (32).

Mixed vitiligo has been reported in a subset of SV patients (7). In our experience, depigmented areas are in the majority of patients limited, but progression to extensive vitiligo vulgaris is possible. Usually, SV precedes the development of NSV (7, 33). Halo naevi are relatively frequent in SV although variable percentages have been reported in the literature (5.3%-24.2%) (34). The frequency strongly depends on the area of SV involvement (trunk > face) (5). Halo naevi and leukotrichia are strong predictors of progression to mixed vitiligo (OR= 24.8 and 25.7, respectively) (33). The presence of halo naevi in SV is associated with a positive family history of vitiligo and thus linked to the genetic background (33). Several theories for the subsequent development of NSV can be proposed. Healthy individuals carry circulating melanocyte-specific T cells with an anergic phenotype (35). This cell population is believed to be a protective mechanism against the development of melanoma when the balance is shifted from anergic to active. Regulatory T cells (Tregs) are implied in the maintenance of this anergic state. During the pathogenesis of SV, these cells may become activated by losing coinhibitory molecules [e.g. cytotoxic T lymphocyte antigen-4 (CTLA-4)] and gaining a higher T cell receptor affinity. This can lead to the active destruction of naevoid and epidermal melanocytes illustrated by the development of halo naevi and NSV in genetically predisposed patients. Another mechanism can be epitope spreading where immune cells involved in targeting SV melanocytes acquire specificity for naevoid or epidermal melanocytes.

## Cases of Segmental Vitiligo Displaying Additional Skin Disorders

Although the rate of autoimmune disorders is not markedly increased in SV, some interesting cases have been described with other inflammatory and non-inflammatory disorders co-occurring at the same site (**Table 1**). A literature search was performed using Embase and Pubmed databases from conception up to May 10, 2020. All original articles, reviews, and different types of articles including letters to the editors, conference papers, and posters were included. Embase was searched using the terms “segmental vitiligo” OR “segmental vitiligo”/exp. Pubmed was searched using “segmental vitiligo” [All fields] OR “segmental vitiligo” [Mesh]. All types of articles, in any language, were included. Articles without full-text availability were excluded. Articles with both SV and NSV were only included in case results on SV were described in detail. The goal was to include all case reports mentioning comorbid disorders in patients with SV or drug-induced SV. The literature search was done by 2 independent reviewers (RS and MS) and was uploaded with Zotero software. The data were extracted by RS. Overlapping studies were excluded based on the author list and content.

**Table 1:** Segmental vitiligo cases with autoimmune and other comorbidities.

Comorbidity	Age (years)	Authors
**Morphea/Parry-Romberg/lichen sclerosus**		
Morphea en coup de sabre	22	Ubaldo et al. (36)
Circumscribed morphea	10	Lee et al. (37)
Morphea at same body area	10, 10	Dev et al. (38)
Morphea	10	Kim et al. (39)
Segmental morphea	18	Yadav P. et al. (40)
Morphea en coup de sabre	46	Janowska et al. (41)
Parry-Romberg syndrome	46	Wolek et al. (42)
Morphea en coup de sabre	9	Bowen et al. (43)
Lichen sclerosus	41	Weisberg et al. (44)
Linear morphea	21	Bonifati et al. (45)
Parry-Romberg syndrome	11	Creus et al. (46)
**Other autoimmune diseases**		
Alopecia areata, segmental lichen planus	12	Kumar et al. (47)
Lichen striatus	4, 5, 6	Correia et al. (48)
Zosteriform lichen planus pigmentosus	22	Sawatkar et al. (49)
Linear psoriasis	47	Valbuena et al. (50)
Alopecia areata, psoriasis	25	van Geel et al. (10)
Lichen nitidus (and Down’s syndrome)	4	Agarwal et al. (51)
Segmental lichen planus	14	Sardana et al. (52)
**Drug-induced**		
Infliximab for rheumatoid arthritis	46	Carvalho et al. (53)
Infliximab for ulcerative colitis	34	Ryu et al. (54)
Immunotherapy for house dust mite	10	Shin et al. (55)
Isotretinoin	17	Avelar-Caggiano et al. (21)
Interferon alpha and ribavirin	60	Tinio et al. (56)
**Co-occurring benign or malignant pigment conditions**		
Cerci et al. (57)	37	Metastatic melanoma
Tiwary et al. (58)	9	Segmental naevus spilus
Kuruvilla et al. (59)	10	Segmental lentiginosis
Luo et al. (60)	8	Naevus of ota
Hofmann et al. (61)	6	Congenital naevus
**Linked neurologic disorders**		
Singh et al. (62)	13	Encephalitis
Yacubian et al. (63)	3	Rasmussen encephalitis
Jang et al. (64)	56	Schwannoma
**Other**		
Utaş et al. (65)	14	Baboon syndrome
Rajashekar et al. (66)	20	Twenty nail dystrophy
Kandpur et al. (67)	14, 23	Twenty nail dystrophy
Muramatsu et al. (68)	22	Naevoid basal cell carcinoma syndrome
Tay et al. (69)	/	Porencephaly, nasofrontal mucoceles, hypertelorism

In total, 564 and 449 results were found for Embase and Pubmed, respectively. For the Embase search, 3 duplicates were removed based on the same content. Titles and abstracts of all articles were screened. One case was excluded due to doubt about the clinical diagnosis of segmental vitiligo based on the clinical picture. Combining both searches, 38 reports could be included describing patients with SV and a comorbid disorder comprising 21 patients with auto-immune disorders, 5 cases of drug-induced vitiligo, 5 cases with benign or malignant pigment disorders, 3 cases with neurological disorders, and 6 other cases

**Figure 1:**
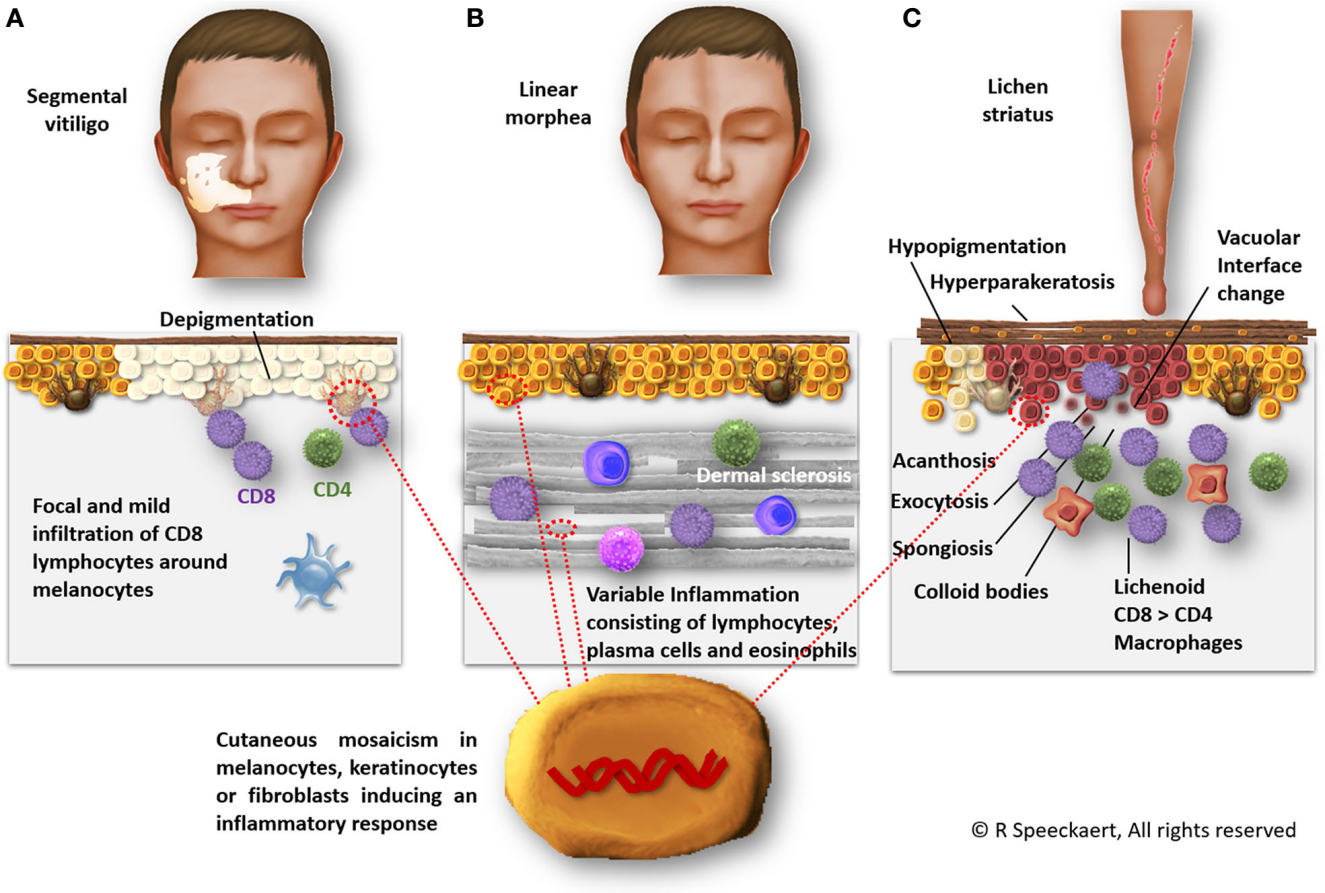
Segmental vitiligo, linear morphea, and lichen striatus are presumed to share a similar pathogenesis based on a genetic mosaicism causing an inflammatory response. In segmental vitiligo **(A)**, a CD8-dominant infiltrate around melanocytes is observed ultimately leading to melanocyte destruction. Linear morphea **(B)** is characterized by dermal sclerosis and an inflammation of variable extent consisting of lymphocytes, plasma cells, and occasional eosinophils. Lichen striatus **(C)** exhibits a focal lichenoid lymphocytic infiltrate with macrophages. Hyperparakeratosis, acanthosis, exocytosis, spongiosis, vacuolar interface dermatitis, and colloid bodies can be observed.

A remarkable number of case-reports with morphea and lichen sclerosus have been published (39, 40, 44, 70). 11 different reports were found which makes it by far the most frequently observed comorbidity [57.1% (12/21 patients) of reported patients with autoimmune comorbidities]. In most cases, the morphea lesions affected the same body area as the SV, although multifocal morphea was also documented (36–46). Cases with lichen striatus are interesting because of the striking similar pathogenesis (48) (**Figure 1**). A cytotoxic lymphocyte attack against postzygotic mutant keratinocytes has been proposed as the underlying mechanism of lichen striatus. This inflammatory response is especially pronounced in the basal and suprabasal layers which explains the development of postlesional hypopigmentation (71). Interestingly, also other localized skin disorders were reported in SV including segmental lichen planus, zosteriform lichen planus pigmentosus, lichen nitidus, and linear psoriasis (48–52). While relatively common in NSV, alopecia areata was only reported in 2 cases (10, 47).

Drug-induced SV is rare, especially infliximab is interesting given the dual effect TNF-α inhibition exerts on NSV (53, 54). TNF-alpha blockers are linked to the new development of NSV whereas also a stabilizing effect on existing NSV has been found (72, 73). A 34-year old patient with ulcerative colitis developed SV 4 months after initiation of infliximab (54). Another 46-year old patient receiving infliximab for rheumatoid arthritis developed SV after 2 months (53). Spreading of pre-existing SV occurred after immunotherapy with house dust mite (55). Finally, interferon-α – a well-known trigger of NSV - induced SV in a patient with hepatitis C (56).

Besides halo naevi, other pigment disorders are rare in SV. Only isolated reports of SV patients with co-existing segmental lentiginosis, segmental naevus spilus, and a naevus of Ota were found (58–61).

Overall, most co-occurring mosaic disorders in SV patients are characterized by a cutaneous inflammation which may be triggered by the immunologic events in active SV (or vice versa, the SV may be induced by the other inflammatory mosaic disorder). Another important group (e.g. segmental lentiginosis, neurologic disorders) consists of genetic or non-genetic diseases affecting melanocytes or their precursor cells.

## Evidence of Somatic Mosaicism

The characteristic distribution patterns of SV suggest a genetically mutated population of melanocytes which is targeted by a cytotoxic cell response. Different patterns of segmental vitiligo in the face and on the trunk have been detected and can easily be recognized in clinical practice (74, 75). By comparing different unilateral and localized skin disorders, we found that the SV pattern does not follow a dermatomal distribution but displays a unique distribution pattern that shares the largest similarity with segmental lentiginosis, followed by epidermal naevus verrucosus (76). Due to the remarkable overlap with segmental lentiginosis, the contribution of other skin cells such as keratinocytes, or alterations in the dermis are less plausible in SV. Additionally, segmental vitiligo follows the embryonal migration lines of melanocyte precursors. During embryogenesis, precursors of melanoblasts migrate from the neural crest in a dorsolateral way. During this migration, an impressive number of cell proliferations takes place enhancing the chance of genetic mosaicism. Another subpopulation of adult melanocytes originates from precursor cells with Schwan cell/melanoblast potential that migrate on a ventrolateral pathway. In mice, 65% of melanocytes of hair follicle melanocytes were linked to this separate group of melanocyte progenitors (77, 78). This might explain why long-lasting perifollicular repigmentation is sometimes possible in SV (14). This reservoir of perifollicular melanocytes and melanocyte precursors is however often not spared after prolonged periods of disease activity and phototherapy early in the disease (<6 months) gives better results (12). Interestingly, the frequency of SV on the back is lower compared to the lateral area, followed by the abdomen which aligns nicely with the migration route of melanoblasts (76). The temporary inflammation followed by a stable course in most SV patients is a clear differentiating factor between SV and NSV. This supports an inherent genetic defect carried by melanocytes in the affected segment. After removal of the susceptible SV melanocytes, the inflammation subsides. This is illustrated by the high success rate of pigment cell transplantations in stable SV compared to NSV (79).

## Neurogenic Theory

For many years, SV was described to follow a dermatomal distribution and consequently, the involvement of neurogenic factors was suspected. Several cases with encephalitis preceding SV have been reported (62, 63). It should, however, be noted that this is also the case for NSV (80). Especially in NSV, elevated levels of neuropeptides [e.g. neuropeptide Y (NPY)] have been discovered. This does not confirm the involvement of neurogenic mechanisms as neuropeptides are also released following skin inflammation in other skin diseases such as psoriasis (81). Nonetheless, neuropeptides control regional skin immunity and some have important effects on melanocytes [e.g. α melanocyte-stimulating hormone (α-MSH)]. 2 cases of SV patient with schwannomas were found. This offers however more a clue toward somatic mosaicism given their common origin from the neural crest than an argument for a neurogenic cause of SV (64). Schwann cells can even *in vitro* be dedifferentiated to glia/melanocyte precursors illustrating their common origin (82). A study including 76 patients with SV found a slower nerve conduction velocity on the lesional side of the body compared to the contralateral side which was more pronounced in stable SV (83). Although the difference was small, it could be shed new light on the neurogenic involvement in SV if these results can be confirmed.

## Future Perspectives

Overall, research on SV remains limited as only 4.8% of vitiligo research discusses findings on SV on Pubmed. This is remarkable as SV is likely to be a less multi genetic disorder compared to NSV which might offer interesting information to the key factors making melanocytes vulnerable for immune-based destruction. The number of publications on SV is even declining in recent years in contrast to the rising yearly numbers on NSV.

Based on previous research, an inflammatory response has been proven in the majority of SV patients. The lack of markedly increased rates of associated systemic autoimmune comorbidities highlights the chance of a susceptible skin environment. As discussed above, the distribution patterns of SV render genetic mosaicism is very likely. This suggests that SV follows a similar pathogenesis compared to lichen striatus, blaschkitis, linear morphea, and linear lupus erythematosus. All these conditions share an inflammatory response in a particular distribution pattern usually at a young age. In most cases, a spontaneous stop of disease activity occurs with limited recurrences during adult life. Lichen striatus has the additional similarity of leaving hypopigmented skin areas which can resolve spontaneously (84). Linear morphea usually presents as a broad linear band of induration with often additional dyspigmentation (both hyper- and hypopigmentation) (85).

However, the underlying genetic mutations have currently not been discovered despite several research projects that have been initiated. This is mainly due to the fact that melanocytes of the inflammatory border of vitiligo are difficult to isolate and proliferate in the lab probably because of their compromised skin environment. Altered E-cadherin expression in SV melanocytes may be a primary deficiency which could be one of the initiating factors of SV hampering the *in vitro* culture of SV melanocytes (86). As the distribution patterns in the face and the trunk have been mapped in detail, future projects could focus on analyzing skin areas that are not (yet) affected but are likely to contain genetically identical melanocytes. Early SV rarely involves the whole skin area which is supposed to be populated by melanocytes originating from the same melanocyte precursor and often leaves skipped areas of normally pigmented skin.

Based on the published case reports, the co-occurrence of (localized) morphea and SV is striking. Although morphea has also been associated with NSV, linear morphea seems particularly linked to SV. Linear morphea follows Blaschko’s lines and genetic mosaicism is therefore believed to be the underlying cause (87). The distribution patterns of SV and linear morphea are clearly different which can be explained by the different skin cell types which are likely responsible for these conditions (melanocytes vs keratinocytes or fibroblasts) (**Figure 1**). Both morphea and SV have been linked to traumata or local enhanced immune responses and therefore one autoimmune condition can elicit the other. It is remarkable that 2 mosaic disorders affecting cell types of different embryogenic origins have a tendency to co-occur in the same body area. Despite some case reports, this phenomenon remains however extremely rare and the differential diagnosis with hypopigmented lesions caused be morphea can be challenging (85).

In conclusion, SV research merits new attention as it may provide interesting data for the pathogenesis and treatment of vitiligo in general. To date, evidence points to a temporary cytotoxic-response targeting mosaic melanocytes. Associated autoimmune diseases are less prevalent compared to NSV, although a remarkable overrepresentation of case reports with (linear) morphea is present. A genetic predisposition (e.g. family history of NSV) may enhance the chance of SV and the subsequent development of halo naevi and NSV. Screening for thyroid disease is less valuable in SV compared to NSV.

## Author Contributions

RS: Conception or design of the work, Data collection, Drafting the article. JL: Critical revision of the article. VB: Critical revision of the article. AB: Critical revision of the article. MS: Critical revision of the article, Data collection. NG: Critical revision of the article. All authors contributed to the article and approved the submitted version.

## Funding 

The research activities of RS and NG are supported by the Scientific Research Foundation-Flanders (Krediet aan Navorsers: 1504718N and FWO Senior Clinical Investigator: 18B2721N and 1831512N, respectively).

## Conflict of Interest

The authors declare that the research was conducted in the absence of any commercial or financial relationships that could be construed as a potential conflict of interest.
